# ShinyCore: An R/Shiny program for establishing core collection based on single nucleotide polymorphism data

**DOI:** 10.1186/s13007-023-01084-0

**Published:** 2023-10-11

**Authors:** Steven Kim, Dong Sub Kim, Hana Moyle, Seong Heo

**Affiliations:** 1 Department of Mathematics and Statistics, California State University, Monterey Bay, Seaside, USA; 2 Department of Horticulture, Kongju National University, Yesan, Korea; 3 Department of Marine Science, California State University, Monterey Bay, Seaside, USA

**Keywords:** Germplasm, Core collection, R/Shiny, Evaluation metrics, Single nucleotide polymorphism

## Abstract

**Background:**

Managing and investigating all available genetic resources are challenging. As an alternative, breeders and researchers use core collection—a representative subset of the entire collection. A good core is characterized by high genetic diversity and low repetitiveness. Among the several available software, GenoCore uses a coverage criterion that does not require computationally expensive distance-based metrics.

**Results:**

ShinyCore is a new method to select a core collection through two phases. The first phase uses the coverage criterion to quickly attain a fixed coverage, and the second phase uses a newly devised score (referred to as the rarity score) to further enhance diversity. It can attain a fixed coverage faster than a currently available algorithm devised for the coverage criterion, so it will benefit users who have big data. ShinyCore attains the minimum coverage specified by a user faster than GenoCore, and it then seeks to add entries with the rarest allele for each marker. Therefore, measures of genetic diversity and distance can be improved.

**Conclusion:**

Although GenoCore is a fast algorithm, its implementation is difficult for those unfamiliar with R, ShinyCore can be easily implemented in Shiny with RStudio and an interactive web applet is available for those who are not familiar with programming languages.

## Background

Germplasms are genetic materials accumulated through evolution and extinction on the Earth. Genetic diversity is essential to breed new cultivars that are agriculturally useful for humans [1, 2]. Genetic resources have been recognized as national properties rather than the shared heritage of humans since the Convention on Biological Diversity in 1992. Therefore, many countries have promoted and preserved the diversity of genetic resources to be utilized as strategic and economic properties. However, with the expansion of genetic resources, the cost and time of their utilization, maintenance, and conservation have increased. Accordingly, core collection, proposed by Frankel [3], has emerged as an alternative. A core collection is defined as a subset of accessions (a sample added to the core collection) that represents the genetic diversity of the entire collection by minimizing repetition and maximizing genetic variation [4–7]. A core collection should have a few redundant entries (an entry is an accession that is selected from the core collection, and an accession is a component of the entire collection), and it should be small enough for convenient use and diverse enough for representing the entire collection [4]. From this broad definition, several operational definitions and criteria have been derived based on different views and specific objectives of the core collection [6, 8, 9]. According to Galwey [10], the purpose of selecting a core collection is to (1) maximize the representativeness of variation trends in the entire collection and (2) maximize the representativeness of the full range of genetic variation in the entire collection. Meanwhile, according to Marita et al. [11], the purpose of selecting a core collection is to (1) maximize the total genetic diversity of the core collection and (2) maximize the representativeness of the genetic diversity of the entire collection.

The quality of a core collection (or simply a core) depends on perspectives and purposes. For instance, Odong et al. [6] classified a core collection into three types, namely CC-D, CC-X, and CC-I. The CC-D type, a concept proposed by Galwey [10] for the first time, selects a core with a similar distribution to the entire collection; therefore, it is likely to include a large number of accessions with common alleles while excluding the rare ones. The CC-D type is preferred by researchers or breeders aiming to select reference sets for various breeding purposes. The CC-X type, a concept put forth by Marita et al. [11], includes accessions in a core with extreme values in both directions of the distribution of the entire collection. Unlike a CC-D core, a CC-X core is likely to select entries with rare alleles; however, it has the disadvantage of adding redundant accessions. Furthermore, accessions located in the middle of the distribution of the entire collection are unlikely to be selected. The CC-X type can be useful for screening resistant or susceptible accessions in a disease-resistance breeding program. Finally, the CC-I type, the second concept proposed by Galwey [10] and Marita et al. [11], includes accessions that represent themselves and similar accessions in the entire collection. Both rare and common alleles are included in a CC-I core, with minimum repetition of similar accessions. To this end, CC-I may be the best approach to construct a core collection for breeders and researchers.

Because the three types of core collections have different perspectives and purposes, each should be evaluated according to different criteria. Odong et al. [6] suggested various distance-based criteria for each of the three types and discussed their properties. First, a CC-X core can be evaluated based on two criteria: the average distance between each entry and the nearest neighboring entry (E-NE) and the average genetic distances between the entries (E-E). A core selected based on E-NE maximizes the average distance between each entry and the nearest contiguous entry to avoid the selection of similar accessions. Meanwhile, the E-E criterion maximizes the average genetic distance between the entries of the core collection, although it may induce high redundancy within the distribution. Thus, the E-NE criterion is more widely used than the E-E criterion for CC-X cores. Next, the evaluation criteria for CC-D were devised to compare the probability distributions of the entire and core collections. In addition to descriptive statistics for the center, spread, and shape, the quantile–quantile plot [12] and Kullback–Leibler distance [13] have been widely used. For categorical data, the chi-square goodness-of-fit can be used as an alternative to measure the discrepancy between the distribution of the core and entire collections. Finally, the average distance between each accession and the nearest entry (A-NE) is used to evaluate the CC-I core. The A-NE criterion selects an optimal core collection by minimizing the average distance between each accession and its nearest entry. The entries tend to be located at the center of clusters rather than at their outer region.

For categorical data, Shannon’s diversity index (SH) [14] and coverage (CV) [15] are commonly used criteria for CC-I [6]. CV measures the proportion of marker alleles in the entire collection that are retained in the core collection [16]. SH measures whether the distribution of marker alleles is well balanced (i.e., equal proportions). Both CV and SH have been devised for CC-I [6], although their aims are different. SH is maximized in the core collection when all possible alleles have the same proportion, although this is not a necessary condition to maximize CV. In the presence of thousands of markers in the core selection, CV serves as an efficient criterion to attain genomic diversity with a few entries; however, researchers and breeders sometimes desire multiple entries of the rarest alleles. Thus, SH is a more suitable criterion. Through examples in the present article, we showed that both CV and SH increase rapidly at the beginning of core selection, but SH stops increasing (or even decreases) mid-way. Furthermore, an increase in the last 1% of CV requires a substantially large number of entries. In this context, considering a two-phase core selection may be reasonable. In the first phase, we focused on CV until the minimum (specified by the user) was attained. In the second phase, we focused on entries with rare alleles. The proposed two-phase core selection is available in R/Shiny, an interactive web applet, for those who are not familiar with the R programming language and is referred to as ShinyCore. In the present study, we compared ShinyCore and GenoCore using two wheat datasets generated with a single nucleotide polymorphism (SNP) array.

## Materials and methods

### GenoCore

For a given core, we defined CV = *m*^− 1^ ∑_*i*_*c*_*i*_ /*e*_*i*_, where *m* is the number of markers, *c*_*i*_ is the number of genotype classes (allele types) for the *i*^th^ marker in the core collection, and *e*_*i*_ is the number of genotype classes for the *i*^th^ marker in the entire collection [5]. This is the average proportion of genotype classes covered by the core across all markers, and this criterion has been devised for the CC-I type [6].

Some samples (accessions from the entire collection) may have missing genotypes. GenoCore determines the first entry with the minimum number of missing genotypes. Once the first entry is determined, GenoCore sequentially maximizes CV until a fixed value is attained (specified by the user). Unlike distance-based measures and SH, CV does not decrease core size (the number of accessions in the core). In other words, an additional entry into the core collection does not lower CV. Compared with other core collection algorithms, which require random processes, GenoCore sequentially maximizes CV, allowing faster computation while selecting the same core (no variations from run to run).

GenoCore attempts to reduce the computation time by calculating the coverage score (which is different from CV) for each accession. The average genotype frequency across all non-missing genotype markers is calculated, and the sample with the highest coverage score, which increases CV at each iteration, is selected [5]. This method rapidly increases CV, because a sample with a high coverage score tends to have more common alleles. When two or more samples have the same coverage score, another statistical measure, the diversity score, can be used [5], and the one with the lowest diversity score is removed.

ShinyCore.

The proposed method, ShinyCore, uses a brute-force method instead coverage and diversity scores to maximize CV. ShinyCore comprises two phases. The first phase, called the covering phase, focuses on maximizing CV. The second phase, called the thickening phase, seeks to include accessions with rare alleles in the final core collection.

ShinyCore does not use coverage score for the following reasons. We argue that two or more accessions with the same coverage score do not imply the same genotype class for all markers. In a high-dimensional dataset (with thousands of markers), the impact of selecting a sample based on coverage and diversity scores is unknown. Therefore, we considered a brute-force approach that maximizes CV at each iteration without eliminating any samples according to coverage and diversity scores. CV calculation in the proposed brute-force approach is expected to be accurate, although this can result in slightly different cores between ShinyCore and GenoCore at the same fixed CV.

During the covering phase of ShinyCore, computation is accelerated after each iteration using the following two operations. First, an NA value is assigned to the marker of an accession if the genotype class of this marker is already covered by the current core. Second, markers are removed such that all genotype classes are fully covered by the current core. The first operation is useful because calculating the number of non-NA genotype classes for each accession is computationally simpler than calculating CV with all possible entries. The second operation reduces the dimension of the working dataset after each iteration, which further shortens computation time, particularly after several iterations.

Researchers and breeders prefer to include more accessions containing the rarest alleles of a marker. For instance, suppose that the entire collection of 100 samples has genotype classes A, B, and C of a marker with frequencies of 5, 45, and 50, respectively. If the core of size 10 includes 1, 1, and 8 of the three genotype classes, this marker is fully covered; however, breeders and researchers may prefer to include more As. Here, SH is the criterion for this purpose (i.e., a uniform distribution of genotype classes). Unlike CV, SH decreases with core size. Thus, the simultaneous maximization of CV and SH can be challenging and computationally expensive.

To address this challenge, we quantified each accession as follows. Suppose that an accession is randomly selected from the entire collection. Then, the probability of selecting the *j*^th^ allele of the *i*^th^ marker can be defined as *η*_*ij*_ = *p*_*ij*_ /(1 − *p*_*ij*_), where *p*_*ij*_ is the proportion (relative frequency) of the *j*^th^ allele of the *i*^th^ marker in the entire collection. Next, we quantified the plausibility of selecting a rare allele as (*η*_*ij*_)^−1^ = (1 − *p*_*ij*_)/*p*_*ij*_, and defined the total rarity score of an accession as RS = ∑_*i*_ (*η*_ij*_)^−1^, where *η*_ij*_ indicates the probability of selecting the j*^th^ allele observed in the *i*^th^ marker for that accession. During the thickening phase, entries with a high RS value are added to increase the likelihood of including rare alleles across all markers.

Core size (the number of accessions in a core) tends to increase exponentially to attain an additional percentage of CV. In other words, substantially more accessions in the core are required to increase CV from 98 to 99% than from 97 to 98%. For some researchers and breeders, given that CV is sufficiently high, including rare alleles in the core collection may be more valuable than increasing the last 1% of CV. Following the covering phase (i.e., after attaining the minimum CV specified by the user), the thickening phase is performed as follows. Let *n*_*p*_ denote the core size required to attain a CV of *p*%. A ShinyCore user may specify the minimum and maximum percent CV as desired. For instance, if a user specifies the minimum CV of 98% and the maximum CV of 99%, ShinyCore works as follows: (1) Covering phase: Find a core of size *n*_*99*_ that attains 99% CV and remembers the core size *n*_*98*_ when it attains 98% CV. (2) Thickening phase: Start from the core of size *n*_*98*_, which attained 98% CV, and then add *n*_*99*_–*n*_*98*_ entries of the highest values of RS from the entire collection that were not selected in the core. The final core size is *n*_*99*_, and the CV is between 98% and 99%.

The interface web application is available at https://stevenkimcsumb.shinyapps.io/ShinyCore. This applet does not require users to understand the R programming language and the R script is available at zenodo repository (10.5281/zenodo.8137684).

## Results

The performance of GenoCore was evaluated based on the CV, SH, and modified Roger’s distance (MR) criteria. The performance of GenoCore has been shown to be superior or comparable to that of the other core selection programs [5]. In this section, ShinyCore is compared to GenoCore using two datasets. The first dataset (hereafter, wheat 1) was derived from the wheat 35 K SNP array dataset developed by Wilkinson et al. [17] (Table 1). The second dataset (hereafter, wheat 2) is part of the wheat 15 and 90 K Infinium array used to analyze quantitative trait loci and perform marker-assisted breeding (MAS) for disease resistance [18] (Table 1).

**Table 1:** Datasets used in the present study

Name	SNP chip	Number of markers	Number of samples	Reference
Wheat 1	Affymetrix Axiom 35 K SNP array	14,099	556	Wilkinson et al. (2012) Jeong et al. (2017)
Wheat 2	15 K & 90 K Infinium array	12,896	890	Wang et al. (2014) Soleimani et al. (2020)

The core of size *n*_*99*_ at the end of the covering phase (before the thickening phase) is referred to as ShinyCore 99% CV. A core of the same size after the thickening process is referred to as ShinyCore 98% CV + T. The core that attained 99% CV using GenoCore is referred to as GenoCore 99% CV; the code is available at https://github.com/lovemun/Genocore [5].

For the wheat 1 dataset, the core sizes of ShinyCore 99% CV, ShinyCore 98% CV + T, and GenoCore 99% CV were 89, 89, and 73, respectively. The core selected by GenoCore 99% CV attained a CV of 99.0% according to the calculation by GenoCore but attained a CV of 98.6% according to calculation by ShinyCore. For the wheat 2 dataset, the respective core sizes were 32, 32, and 28, and GenoCore 99% CV attained a CV of 98.7% according to calculation by ShinyCore.

**Fig. 1:**
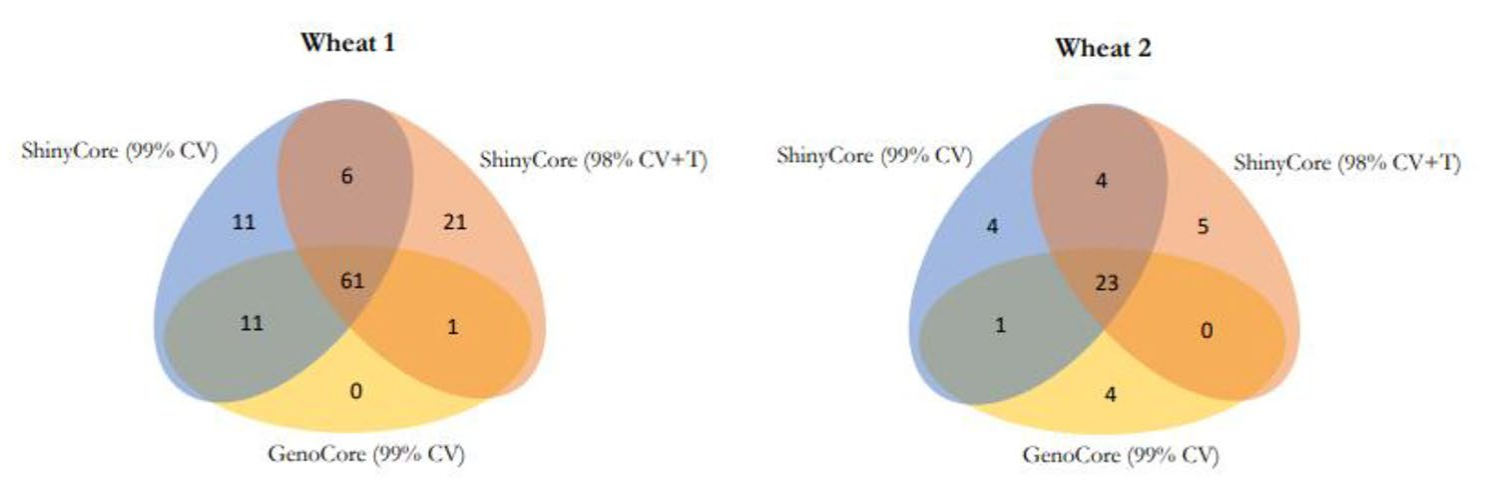
Venn diagrams of accessions selected by ShinyCore (99% CV and 98% CV + T) and GenoCore (99% CV). The diagrams indicate wheat 1 (left) and 2 (right) datasets, respectively

Venn diagrams of the three core collections are presented in Fig. 1. The three cores shared a large number of entries. Despite the different approaches for reducing the computation time between GenoCore and ShinyCore, 72 of the 73 entries in GenoCore 99% CV overlapped with those in ShinyCore 99% CV for the wheat 1 dataset. ShinyCore attained a CV of 98% with *n*_98_ = 55 entries, and none of the additional entries of ShinyCore 98% CV + T overlapped with those of ShinyCore 99% CV. Therefore, the covering and thickening phases were markedly different. A similar trend was observed for the wheat 2 dataset.

Table 2 summarizes the cores of GenoCore 99% CV, ShinyCore 99% CV, and 98% CV + T for the wheat 1 and 2 datasets. We observed similar trends for both datasets as follows. ShinyCore formed a larger core size than GenoCore at the same fixed CV. The core selected by GenoCore 99% CV showed a slightly lower CV than 0.99 according to calculation by ShinyCore. ShinyCore attained a CV of 99% faster than GenoCore. For the thickening phase, ShinyCore 98% CV + T required an additional computation time of 0.19 and 0.16 min for the wheat 1 and 2 datasets, respectively. Finally, the thickening phase of ShinyCore 98% CV + T enhanced genetic diversity (as measured by SH) and genetic distance (as measured by MR) at the cost of a slight decrease in CV.

**Table 2:** Comparison of evaluation metrics between ShinyCore and GenoCore

Dataset	Software	Core size	CV^a^	SH^b^	MR	Time (min)^c^
Wheat 1	GenoCore (99% CV)	73	0.986	0.632	0.604	3.45
	ShinyCore (99% CV)	89	0.990	0.626	0.601	0.59
	ShinyCore (98% CV + T)	89	0.987	0.644	0.608	0.78
Wheat 2	GenoCore (99% CV)	28	0.987	0.768	0.711	1.66
	ShinyCore (99% CV)	32	0.990	0.770	0.711	0.53
	ShinyCore (98% CV + T)	32	0.987	0.777	0.714	0.69

^a^CV was calculated using ShinyCore and was slightly lower than the value reported by GenoCore.

^b^SH was calculated using the logarithm of base *c*, where *c* is the number of genotype classes of each marker, and this base gives SH values between 0 and 1, with 1 implying equal proportions

^c^Computation time was calculated using the same computer (16.0 GB; 10th generation of Core i5)

Regarding the shortened computation time, using the same computer (16.0 GB; 10th generation of Core i5), ShinyCore 99% CV was approximately 5.8 and 3.1 times faster than GenoCore 99% CV for the wheat 1 and 2 datasets, respectively. We anticipate a substantially reduced computation time when a larger dataset is analyzed.

**Fig. 2:**
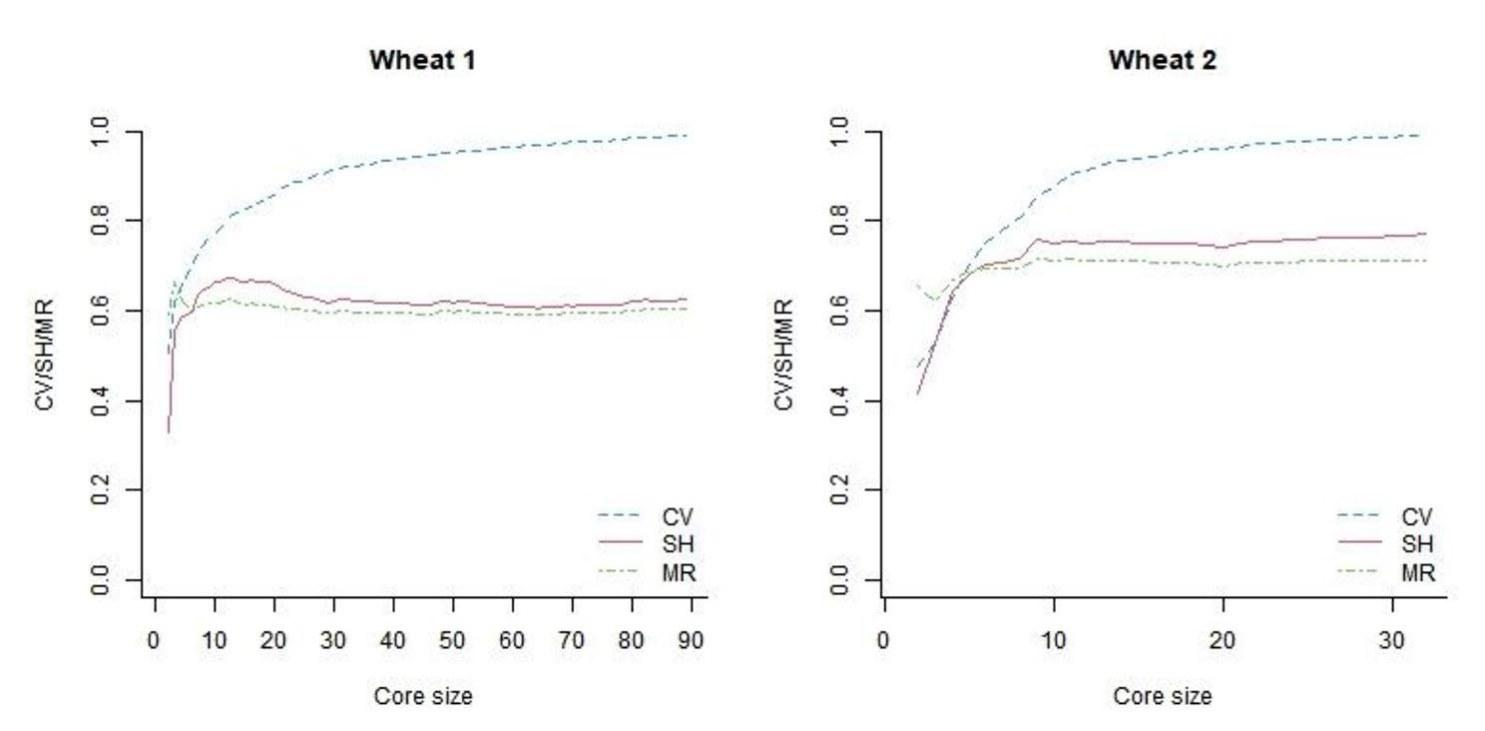
Coverage (CV), Shannon’s diversity index (SH), and modified Roger’s distance (MR) with respect to core size of wheat 1 (left) and 2 (right) datasets. This figure is drawn based on the core selected by ShinyCore 99% CV

Figure 2 shows CV, SH, and MR with respect to core size using ShinyCore 99% CV. SH was calculated using the logarithm of base *c*, where *c* is the number of genotype classes for each marker. This helped visualize CV, SH, and MR simultaneously on a single graph, as the values of all three measures range between 0 and 1, with 1 indicating the most plausible value for each criterion. CV uniformly increased with respect to core size, but the SH and MR did not. SH increased up to a certain core size and then plateaued or decreased. MR did not change significantly with respect to core size. Therefore, even if we increase the core size using GenoCore, the values of SH and MR are unlikely to be greater than those obtained using ShinyCore. When ShinyCore 98% CV + T was compared to GenoCore 99% CV, the calculated CV was the same for both wheat datasets, but ShinyCore 98% CV + T selected a larger core that was genetically more diverse and distant than GenoCore 99% CV (Table 2). Since ShinyCore seeks to include accessions with rare alleles in the thickening phase, it may include more diverse entries than GenoCore, which is advantageous in genome-wide association studies.

**Fig. 3:**
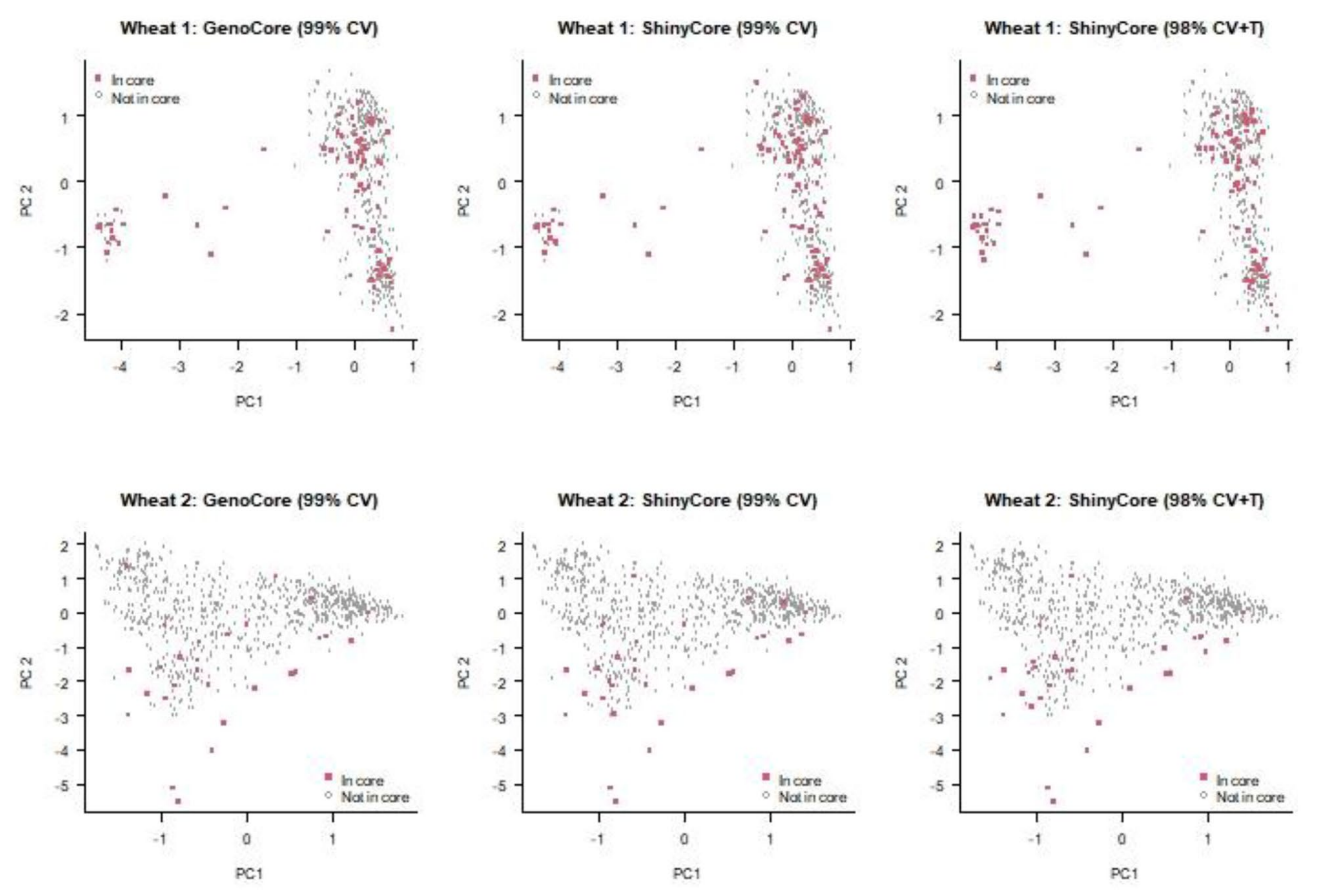
Principal component analysis (PCA) biplots showing entries included in the core subset (red square) and accessions of the entire collection (white circle). The top and bottom three panels represent the wheat 1 and 2 datasets, respectively

Figure 3 presents the results of principal component analysis (PCA) applied to compare the three cores selected by GenoCore 99% CV, ShinyCore 99% CV, and 98% CV + T. In the two-dimensional space of PC1 (x-axis) and PC2 (y-axis), the entries are well spread and the locations of the three cores are similar. For the wheat 1 dataset (the top three panels of Fig. 3), ShinyCore 98% CV + T included more extreme entries in the bottom-right clusters (high values of PC1 and low values of PC2), which were excluded from the other two cores. From a distance-based perspective, these extreme entries may be considered redundant. In contrast, ShinyCore 99% CV included an extreme entry with the maximum value of PC2 (the top middle panel of Fig. 3), which was excluded from the other two cores. For the wheat 2 dataset (the bottom three panels of Fig. 3), GenoCore 99% CV included an entry with the maximum value of PC2 (the top left panel of Fig. 3), which was excluded from the two cores selected by ShinyCore. Overall, GenoCore 99% CV selected the most diverse core among based on the results of two-dimensional PCA, although its SH and MR values were lower than those of ShinyCore 98% CV + T (Table 2). The PCA plot provides an overall graphical assessment and comparison; however, it is limited in its ability to evaluate genomic diversity and distance, because a large amount of information is lost when thousands of markers are summarized into a two-dimensional space.

**Fig. 4:**
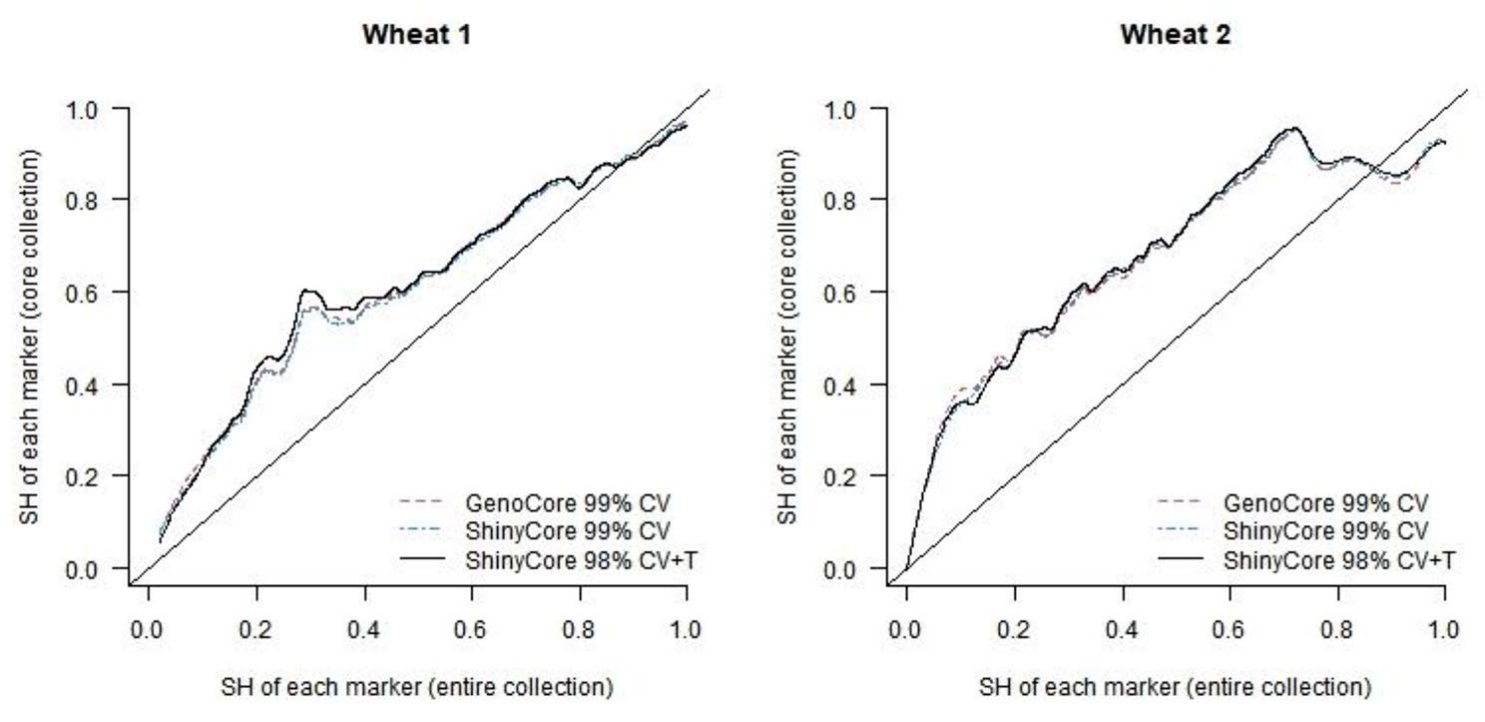
Shannon diversity index (SH) of the core collection for each marker of wheat 1 (left) and 2 (right) datasets

**Fig. 5:**
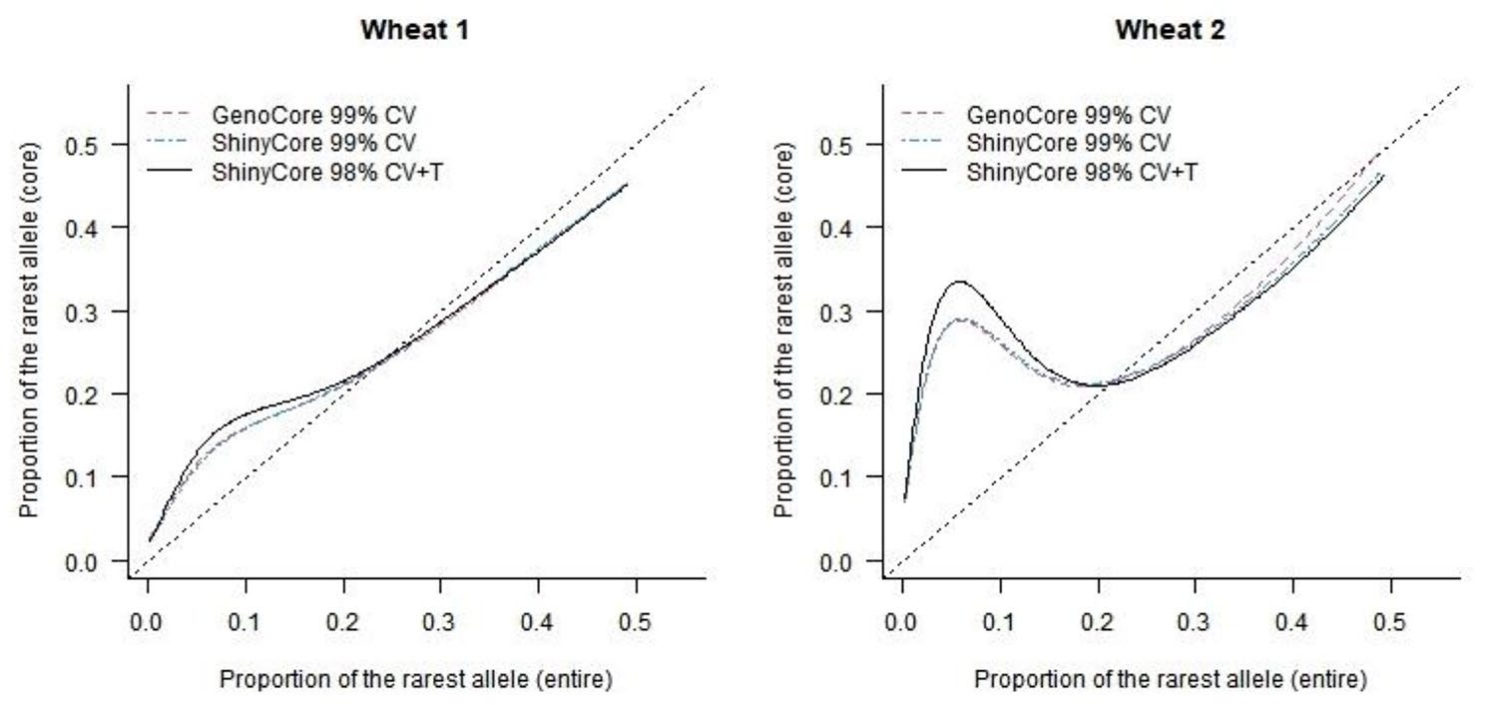
Comparison of the proportion of the rarest allele of each marker in wheat 1 (left) and 2 (right) datasets (x- and y-axes presents the proportions observed in the entire and core collection, respectively)

As an alternative, we visually evaluated the effects of the thickening phase of ShinyCore, as shown in Figs. 4 and 5. We calculated the SH value of each marker for the entire collection and each core. In Fig. 4, the x-axis represents the SH value of each marker in the entire collection, and the y-axis represents the SH value of each marker in the core. The black solid curve represents the SH value of ShinyCore 98% CV + T with respect to the SH value of the entire collection, and it is mostly above the red and blue dotted curves (GenoCore 99% CV and ShinyCore 99% CV, respectively) for both datasets. For the wheat 1 dataset, ShinyCore 98% CV + T, ShinyCore 99% CV, and GenoCore 99% CV showed the maximum SH values of 45.9%, 18.9%, and 35.2% for 14,099 markers, respectively. For the wheat 2 dataset, the respective SH values were 42.4%, 30.4%, and 27.2% for 12,896 markers. Therefore, the thickening phase is indeed helpful, particularly in balancing the distribution of severely unbalanced alleles.

Each marker showed the rarest allele, and the proportion of the rarest alleles could be calculated for each marker in the entire and core collections. In Fig. 5, the x- and y-axes represent the proportion of the rarest alleles of each marker in the entire and core collection, respectively. From the perspective of CC-I type, which is the primary objective of GenoCore and ShinyCore, a high proportion of the rarest allele in the core is plausible, particularly when their proportion is low in the entire collection. Here, all three cores demonstrated a plausible property, and ShinyCore 98% CV + T appeared to be the most suitable in this regard.

## Discussion

Indeed, the thickening phase helps increase SH and MR, with a slight loss of CV. The required core size increases exponentially with CV, which allows the users to specify the minimum and maximum value. The two control parameters can be used to temper the inclusion of rarer alleles. If the minimum CV is set at a lower value (at a fixed value of the maximum CV), the resulting core will include more rare alleles (according to the rarity score) at the cost of a slight loss of CV value (Table 3). ShinyCore has been designed such that (1) its final core size is the same as the core size at the maximum CV, (2) its final CV is guaranteed to be between the minimum and maximum, and (3) it seeks more genetic diversity and distance after attaining the minimum CV. ShinyCore achieves the same goal as GenoCore within a shorter time. In addition, ShinyCore forms two cores—one after the covering phase (before the thickening phase) and the other after the thickening phase; therefore, users can select any of the two. As a future direction, we may consider more parameters to temper the inclusion of more rare alleles (e.g., different kinds of rarity score) and make the various options available as an R package. For users who are not familiar with programming, the menu-driven ShinyCore applet is available which is simple and easy to use (see supplemental data and materials).

**Table 3:** The core metrics (CV, SH, and MR) and the logarithmic rarity score, log(RS), with respect to the minimum coverage (%) at the maximum coverage of 99% specified by user

	Specified Parameters		Core Metrics (CV + T)			
Dataset	Maximum CV (%)	Minimum CV (%)	CV (%)	SH	MR	log(RS)
Wheat 1	99	98	98.7	0.644	0.608	6.710
	99	97	98.6	0.643	0.607	6.712
	99	95	98.6	0.641	0.606	6.714
	99	90	98.5	0.643	0.607	6.715
Wheat 2	99	98	98.7	0.777	0.714	6.795
	99	97	98.6	0.777	0.714	6.816
	99	95	98.4	0.777	0.714	6.834
	99	90	97.9	0.778	0.714	6.851

The choice of a criterion in core selection depends on the breeders’ and researchers’ perspectives and objectives [6], and they tend to value multiple criteria instead of a single one. In the present study, we focused on coverage (as measured using CV) as well as genetic diversity and distance (as measured using SH and MR). However, maximizing all three criteria simultaneously is challenging, and a trade-off is inevitable. Alternatively, a weighted criterion can be considered after normalizing multiple criteria [19, 20]. In future studies, the advantages and disadvantages of weighted criteria (simultaneous optimization) versus a sequential approach (multi-phase optimizations or one criterion per phase) should be explored. Finally, many core selection programs are available, each devised with specific objectives, criteria, and computational methods and each with certain advantages and disadvantages. Therefore, a fair comparison among multiple core selection programs is often difficult. In the present study, we compared ShinyCore and GenoCore, as their objectives and criteria were very close. Users should make practical decisions.

## Conclusion

The size of the entire collection has been increasing in genetic diversity analyses; therefore, computation time has become a critical component of a core selection program. In the present study, we focused on the CC-I type of core selection, whose quality can be evaluated based on both coverage and diversity. We accelerated the covering phase and developed and implemented the rarity score of individual accessions to accelerate the thickening phase. Similar to GenoCore, ShinyCore uses sequential optimization, which not only renders the algorithm fast but also attains a consistent core across runs.

## Data Availability

ShinyCore is available in two versions. The simple version is available at https://stevenkimcsumb.shinyapps.io/ShinyCore1, and the other version is available at https://stevenkimcsumb.shinyapps.io/ShinyCore2. Comparing to the simple version, the other version has additional graphics, and it takes longer computation time than the simple version. In case when the dataset is too large to be analyzed in the server, users can download either version which are available at GitHub (https://github.com/heoseong/ShinyCore) and Zenodo repository (10.5281/zenodo.8137684).
